# Correlation between Dental Vestibular–Palatal Inclination and Alveolar Bone Remodeling after Orthodontic Treatment: A CBCT Analysis

**DOI:** 10.3390/ma12244225

**Published:** 2019-12-16

**Authors:** Cinzia Maspero, Francesca Gaffuri, Iury O. Castro, Valentina Lanteri, Alessandro Ugolini, Marco Farronato

**Affiliations:** 1 Department of Biomedical, Surgical and Dental Sciences, School of Dentistry, University of Milan, 20100 Milan, Italy; cinzia.maspero@unimi.it (C.M.); valentina.lanteri@unimi.it (V.L.); marco.farronato@unimi.it (M.F.); 2 Fondazione IRCCS Cà Granda, Ospedale Maggiore Policlinico, 20100 Milan, Italy; 3 Department Orthodontics, School of Dentistry, Universidade Federal de Goiás, Goiania 74605-220, Brazil; iurygo@hotmail.com; 4 Orthodontic Section, Department of Sciences Integrated Surgical and Diagnostic, University of Genova, 16132 Genova, Italy; alexugolini@yahoo.it

**Keywords:** cone-beam computed tomography, alveolar bone loss, bone remodeling, tooth movement, torque

## Abstract

The aim of this study was to evaluate the correlation between dental vestibular–palatal inclination changes and the cortical bone remodeling after fixed orthodontic treatment using cone beam computed tomography (CBCT). Twenty-two patients with Angle Class I malocclusion, permanent dentition, and mild to moderate dental crowding were included in the present three-dimensional (3D) analysis. Bone dimensions were evaluated by CBCT scans obtained before and after orthodontic treatment, whereas the torque values were calculated by means of digital models using the 3D VistaDent software. A paired t-test was used to compare the changes between the pretreatment and post-treatment measurements. The correlations between variables were analyzed with linear regression analysis. A significant correlation between torque variations and bone thickness changes was observed for the apical buccal level of the anterior side (*P* < 0.05). Limited and not significant alveolar bone resorption for the apical thickness of anterior teeth occurred at ± 5 degrees of torque variation, while for tooth inclination exceeding +5 or −5 degrees, the bone remodeling was more evident. The present study demonstrated that anterior region was the most affected area by bone remodeling and that torque variation was highly related to apical bone thickness adaptation for maxillary and mandibular incisors and maxillary canines.

## 1. Introduction

Orthodontic tooth movement is the result of resorption and apposition of the alveolar bone due to the application of controlled forces on teeth [1].

A basic concept in orthodontics is the “Bone Traces Tooth Movement”, which refers to the strong correlation between orthodontic tooth movement and surrounding bone remodeling, together with the stretching of periodontal ligament fibers [2]. Many studies have observed the mechanical adaptation of alveolar bone in response to orthodontic forces, resulting in resorption and new bone formation in the area of pressure and tension, respectively [3]. The reaction of periodontal tissue depends on the width, height, and morphology of the root, dimensions, angulation, and spatial position of the tooth. Furthermore, it has been shown that bone reaction to orthodontics forces is highly affected by the patient’s bone anatomy, physiology, and adaptability [3].

The thickness of alveolar bone may represent a limit to orthodontic movement since exceeding it can cause undesirable side effects for the periodontal tissues such as attachment loss ad gingival recession. The most critical mechanics, such as dental arch expansion and incisor buccal-lingual movements, can decentralize teeth from the alveolar bone envelope [4]. 

Significant bone dimensional changes occur even with small amounts of tooth movement induced by fixed appliances in patients with mild or moderate crowding. The suitable teeth placing in the center of the alveolus is considered a key factor for the stability of orthodontic results [5]. Therefore, a pretreatment alveolar bone dimensions evaluation [6] may help the orthodontist in detecting the limits of tooth movement, reducing at the same time the risk of iatrogenic bone fenestration or dehiscence [7].

Bone thickness has been traditionally evaluated with lateral cephalograms, panoramic, and/or periapical radiographs [8]. However, the limits of two-dimensional (2D) imaging have been widely demonstrated [9,10]. Among these, distortion, errors of identification, super-imposition, and reduced measurement accuracy are considered the main disadvantages [9,10,11].

On the other hand, three-dimensional (3D) cone beam computed tomography (CBCT) has specifically been introduced and successfully used to evaluate the bony architecture [12,13,14,15,16,17]. Its advantages include, but are not limited to low radiation dose, lower cost, high spatial resolution, excellent tissue contrast, the elimination of blurring and overlapping of adjacent teeth, and overall much more precise cephalometric analyses [18,19]. Timock et al. investigated the accuracy and reproducibility of CBCT in evaluating alveolar bone height, demonstrating its great reliability, especially for the buccal bone height [6,20,21,22]. In addition, CBCT has been also successfully used for determining the risk level of bone loss prior to orthodontic treatment based on dentoalveolar bone phenotypes [23] or tooth position [24]. 

The examination of cortical bone variations induced by orthodontic treatment has traditionally been a critical research topic [25].

Zachricsson et al. demonstrated that orthodontic patients had significantly greater alveolar bone loss than untreated subjects [25], while Polson and coworkers failed to find any detrimental long-term effects of orthodontic treatment on the alveolar bone level [26].

However, given the superior accuracy of three-dimensional methods in identifying bone volume, alveolar bone remodeling due to orthodontic treatment and the amount of bone changes in relation to the dental torque variation should be investigated with CBCT analysis.

Therefore, the aim of this study was to evaluate the correlation between dental torque changes and the amount of cortical bone thickness and height changes after fixed orthodontic treatment using CBCT.

## 2. Materials and Methods 

This study included records selected by a retrospective screening of CBCT images archived at the Federal University of Goiás, Brazil between 2011 and 2014. 

In a previous study [27], 22 patients were selected to calculate the reliability, and the sample size indicated the need for at least 26 patients to estimate vestibulo–palatal inclination with the confidence interval (95%), maximum error of 2.3°, and a standard deviation of 5.9° with a power of 80%.

The 3D images were obtained using an I-CAT CBCT (Imaging Sciences International, Hatfield, PA, USA) configured for 0.25 mm volumetric reconstruction, an isometric voxel, 120 kVp tube voltage, a field of view (FOV) of 13 cm, a 3.8 mA tube current, and an exposure time of 40 s. The high definition and sensitivity of CBCT scans ensures that the buccal and palatal/lingual cortical bone, together with teeth, is visualized without any overlapping.

The inclusion criteria for the sample selection were as follows: a mean age of 13 years (range 11–16) at the baseline, Angle Class I malocclusion, permanent dentition, and mild to moderate dental crowding. The crowding was digitally quantified using the maxillary and mandibular Little irregularity index. Data were collected by a single observer (F.G.) and checked by another examiner (I.C.) to estimate the reproducibility of the process.

The Little index measures the distance between the contact points of rotated teeth, and then adds them together. Therefore, the Irregularity index is the sum of all the displaced contacts between the anterior teeth (canine to canine). 

Records showing decayed teeth, deciduous dentition, impacted molars, periodontal disease, traumatic dental injury, and metal restorations were excluded.

The data of 22 subjects (nine males, 13 females) obtained before and after orthodontic treatment for diagnosis and treatment plans were included in our sample. 

The straight-wire technique, Roth prescription [28], slots 0.022 × 0.028 mm, had been used for their orthodontic therapy, and the outcomes were reported to be in agreement with Andrews’s six keys of normal occlusion [29]. The arches sequence was 0.012 NiTi, 0.014 NiTi, 0.016 NiTi, 0.016 stainless steel, 0.018 stainless steel, 0.017 × 0.025 NiTi, 0.018 × 0.025 titanium molybdenum alloy and 0.019 × 0.025 stainless steel.

The average treatment time was 22 ± 4.2 months, and the subjects were reviewed at 4-week intervals. No patients underwent extractions or palatal expansion during the treatment period.

Patient data were handled according to the requirements and recommendations of the Declaration of Helsinki. Informed consents have been obtained from the former patients.

This study was approved by the Research Ethics Committee of the institution where the CBCT scans were performed (Brazil Platform, Federal University of Goiás, Brazil #024439/2014). 

### 2.1. Evaluation of Crown Inclination

All the plaster models were scanned in centric occlusion using a 3D desktop scanner (3Shape R700, 3Shape A/S, Copenhagen, Denmark) and the standard triangle language (STL) files were imported into the 3D software VistaDent (Dentsply, New York, NY, USA). 

Reference points were marked on the surface of the digital models to define the occlusal plane and landmarks for each tooth. The planes were automatically generated by the software to measure crown inclination (CI), which was defined as the angle between a line perpendicular to the occlusal plane and a line that was parallel and tangent to the vertical axis of the clinical crown [30]. 

CI was considered positive when the occlusal portion of CI line was more buccal at the gingival portion and negative when it was more palatal/lingual.

### 2.2. Measurements of Bone Height and Thickness

**Figure 1:**
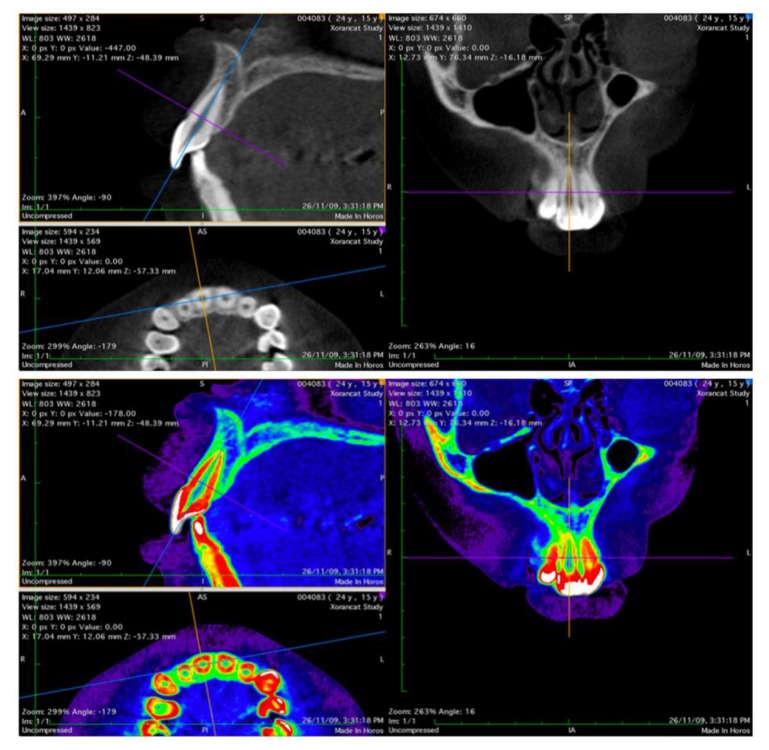
Horos 3.0 software (Horos Project, Annapolis, MD, USA): DICOM medical images viewer.

Vertical alveolar bone distances and horizontal thicknesses around the maxillary and mandibular central and lateral incisors, canines, first and second premolars, first molars, at the baseline and post orthodontic treatment, were measured by the same examiner (F.G.). The images were analyzed using Horos 3.0 software (Horos Project, Annapolis, MD, USA), and they are presented in Figure 1.

**Figure 2:**
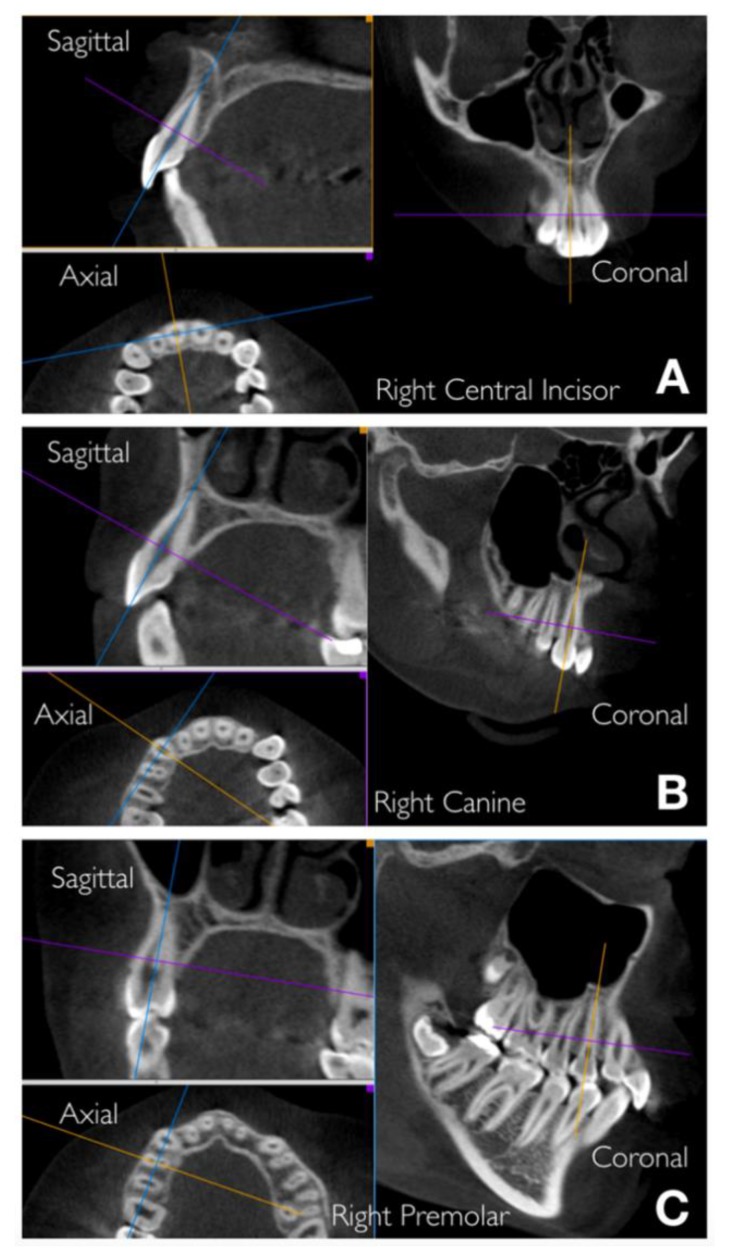
Axial-guided navigation (AGN) method to achieve an optimal visualization of the marginal bone moving the axial cursor on the sagittal or coronal multiplane reconstructions. Examples: (**A**) upper right central incisor, (**B**) upper right canine, (**C**) upper right premolar.

Axial-guided navigation (AGN) was used to locate landmarks [31]. This method was named AGN because all the measurements, which are provided in millimeters, were made by moving the axial cursor on the sagittal or coronal multiplane reconstructions guided by the axial plane along the axis of the dental root to achieve an optimal visualization of the marginal bone in the chosen view (Figure 2). 

**Figure 3:**
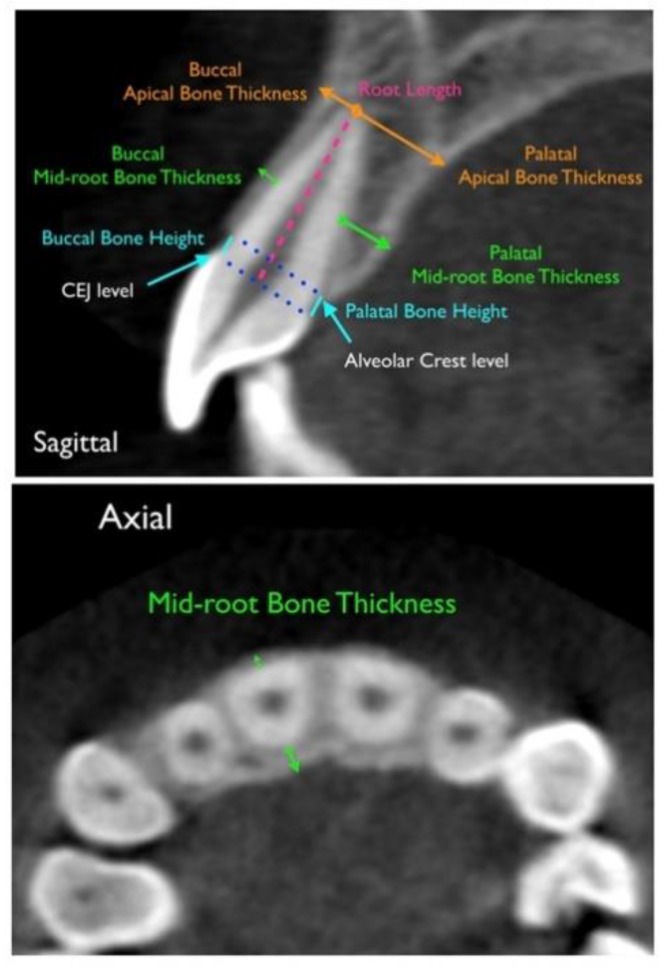
Reference lines and measurements in the sagittal and axial view for an upper central incisor.

Reference points, lines, and dimension variables are described in Figure 3.

To determine which slice should be used for measuring the distance between the cement–enamel junction (CEJ) and the alveolar bone crest (AC), the sagittal section following the vertical dental axis (VDA) was selected for anterior teeth, whereas the coronal section orthogonal to the tooth axis was considered for posterior teeth. For single-rooted premolars, VDA was determined according to the cusp tip and the root apex, while for two-rooted premolars, the reference points were the buccal cusp tip and the apex of the buccal root; lastly, for molars, the reference points were the mesiobuccal cusp tip and the mesial root apex.

Buccal bone height (BHb) and palatal/lingual bone height (BHp) indicated that the AC–CEJ distances were measured parallel to the long axis of the tooth. These measurements represent the amount of vertical alveolar bone loss [32]. The buccal (aBTb, mBTb) and palatal/lingual (aBTp, mBTp) bone thicknesses were measured at the mid-root and root apex level, perpendicularly to the long axis of the tooth. In order to have a more precise evaluation of the thickness values, we controlled each measure of anterior teeth both in the coronal and in the sagittal slices. Furthermore, to analyze apical root resorption in all teeth, the distances (the root length) between the dental apex and the incisal edges or buccal/lingual cusp tips (depending on taking in consideration single or two-rooted teeth) were measured with images generated according to the maximum size zoom of the tooth. Table 1 contains the definitions of all the abbreviated measurements.

### 2.3. Statistical Analysis

Data were collected by the principal investigator (F.G.) and checked by a second examiner (I.C.) to evaluate the reproducibility of the method. Measurements and landmark locations were repeated (10 days after the first measurement) on 20 randomly selected CBCTs for the variables BHb and BHp. The Dahlberg’s values method error was performed, and intra-class correlation coefficients were calculated.

The Shapiro–Wilks test showed that data were normally distributed. A paired t-test was used to compare the changes between the pretreatment and post-treatment measurements. The correlations between variables were analyzed with linear regression analysis. Probabilities of less than 0.05 were accepted as significant in all statistical analyses (*P* < 0.05).

## 3. Results

Dahlberg’s values method error ranged from 0.12 mm to 0.25 mm (not significant). Intra-class correlation coefficients values were larger than 0.92. Standard deviations between repeated measurements were found to be in the range of 0.09 to 0.21 mm for all measurements. Overall, the method error was considered negligible. 

Twenty-four teeth per patient amounting to a total of 528 teeth were included in the analysis. Table 2 and Table 3 depict the statistically significant changes observed after treatment compared to the baseline for all the variables measured. 

Linear regression analysis was performed to detect the associations between variables and torque variation before and after treatment (Table 4 and Table 5). 

The results showed significant correlations between torque variation and the following variables (*P* < 0.05): aBTb for incisors and canines, aBTp for incisors and maxillary canines, mBTb for maxillary lateral incisors, maxillary canines, and mandibular incisors, and mBTp for mandibular central incisors. BHb, BHp, mBTb, mBTp, and RootL were not significantly correlated with torque changes.

**Figure 4:**
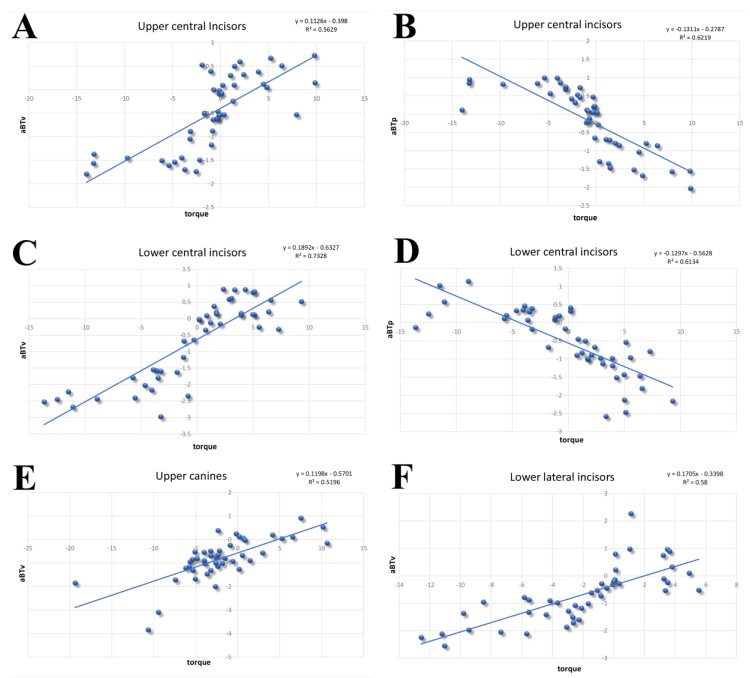
Linear regression model of the six most statistically significant analysis (**A**) maxillary central incisors, aBTb; (**B**) maxillary central incisors, aBTp; (**C**) mandibular central incisors, aBTb; (**D**) Mandibular central incisors, aBTp; (**E**) mandibular lateral incisors, aBTb; and (**F**) maxillary canines, aBTb.

The linear regression model showed threshold values of apical bone thickness resorption (Figure 4).

With torque variations between ±5 degrees, maxillary central incisors showed bone remodeling ranging from a resorption of 1.5 mm to an apposition of 0.5 mm in the buccal sides and from −1.5 mm to +1 mm in the palatal sides. Similarly, torque changes of ±5 degrees seem to produce an apical alveolar bone loss up to −1.5 mm for the lingual sides in mandibular central incisors. A greater variability was found in the buccal aspect where alveolar bone thickness underwent up to 2.5 mm of bone loss and apposition of up to 0.5 mm. 

Lower lateral incisors had an apical facial bone remodeling ranging from −2 mm to +0.5 mm for torque variations between 0 and −4 degrees. Maxillary canines seemed to be less affected by torque changes: for ±5 degrees variations, the buccal bone loss, at the apical level, was −1.5 mm to 0.

## 4. Discussion

Bone remodeling is a constant feature following orthodontic treatment [33]. Our results showed that the mandibular anterior region is the most affected area by bone remodeling. Indeed, the variable bone thickness (aBTp, aBTb) was highly associated with torque variation for all the incisors and maxillary canines.

Orthodontic treatment can lead to marginal alveolar bone loss and gingival recession, as demonstrated in some studies. Furthermore, patients presenting lingual-inclined incisors have lower aBT values at the root apex of the maxillary central incisor than patients with normal and labial-inclined incisors, and they appear to be more susceptible to bone defects [34]. 

Based on CBCT, several studies were able to demonstrate greater marginal bone loss in patients who had undergone orthodontic treatment compared to untreated subjects [35]. However, the magnitude of this bone remodeling is still to be determined. 

In a CBCT study measuring the alveolar bone thickness around maxillary incisors, Tian et al. showed that tooth inclination affects the bone dimensional changes following treatment [34]. In particular, lingual-inclined incisors were found to be more related to bone defect, with fenestrations being more prevalent on the labial side. The authors highlighted the importance of a preliminary comprehensive evaluation of preexisting bone prior to orthodontic treatment [34]. 

Aass and Gjermo [36] found a marginal bone loss of >2 mm in 16.2% of orthodontically treated 14-year-old subjects and in 4.3% of untreated subjects. Moreover, Fuhrmann et al. [4] showed a high frequency of dehiscences and fenestrations at mandibular incisors in a computed tomography study of 11 adult patients undergoing orthodontic treatment. They concluded that radiographic imaging free from superimposed structures permits marginal bone assessments at surfaces not clearly visualized by conventional radiography.

However, to the best of our knowledge, no human studies have investigated the relation between dental torque and bone changes for each tooth location yet. The biological effectiveness of alveolar bone remodeling is due to the periodontal ligament’s need to maintain its width unchanged. The tooth moves through the alveolar process carrying with him his alveolus, with the movement that occurs through the alveolar bone (heavy forces) or with itself (light forces) [3,37]. 

Bucco-lingual movement of the anterior teeth intending to improve the sagittal relationship of the maxillary and mandibular arches is often mandatory to achieve a harmonious profile. However, excessive tooth movement can cause iatrogenic sequelae, including root resorption, gingival recession, and alveolar bone loss. 

This study focused on the remodeling pattern of the alveolar bone of maxillary and mandibular arches, considering the torque variation between the pretreatment and post-treatment. 

In agreement with Zhou et al. [38], the present study demonstrated a significant correlation between the torque variation and buccal bone thickness at the root apex. Limited bone resorption for the apical thickness of the anterior teeth was found for torque variation of ±5°, while for tooth inclination exceeding +5 or −5°, the bone remodeling was more pronounced.

Appropriate tooth torque is important for achieving better occlusion, facial esthetics, and stability.

It can be speculated that when orthodontic movement occurs in the certain range (±5° of torque), alveolar bone is able to withstand the stress of the induced forces without resulting in a significant bone loss, while for inclination exceeding a certain limit (+5° or −5° according to our results), the hard tissues remodeling is more unpredictable and potentially harmful to the buccal anterior teeth “wall”.

These results highlight the importance of dental torque on bone remodeling. Orthodontic movements exceeding 5° on lower central incisors are considered to be more at risk of developing pronounced bone resorption than others. 

Among the limitations of the present study, it should be mentioned that all the enrolled patients who underwent orthodontic fixed therapy were Angle Class I malocclusion with mild to moderate crowding; therefore, we cannot speculate on bone remodeling for other malocclusions and for severe teeth crowding. Lastly, the limited sample size prevented investigating the average bone loss for torque variations exceeding +5 or −5°. 

## 5. Conclusions

The present study shows a specific and detailed method that is able to measure for all teeth many variables and parameters that are key points for any orthodontic treatment.

Our results showed that the most relevant bone remodeling occurred in the mandibular anterior region, and that there is a significant correlation between dental torque changes and bone thickness remodeling for maxillary and mandibular incisors and maxillary canines after fixed orthodontic treatment. These findings should be considered when planning orthodontic treatment. 

## Figures and Tables

**Table 1:** List of abbreviations.

*Measurements*	*Definitions*
*aBTb*	*buccal apical bone thickness*
*aBTp*	*palatal apical bone thickness*
*BHb*	*buccal bone height*
*BHp*	*palatal bone height*
*mBTb*	*buccal mid-root bone thickness*
*mBTp*	*palatal mid-root bone thickness*
*RootL*	*root length*

**Table 2:** Mean values of the variable differences between pre- and post-treatment measurements for the maxillary arch.

*Teeth (maxilla)*	*Central Incisors*		*Lateral Incisors*		*Canines*		*Premolars*		*First Molars*	
	*Mean ± SD*	*P*	*Mean ± SD*	*P*	*Mean ± SD*	*P*	*Mean ± SD*	*P*	*Mean ± SD*	*P*
***∆Tq***	*−0.8 ± 5.2*	*0.334*	*−0.8 ± 5.4*	*0.329*	*−2.0 ± 5.3*	0.000 ***	*−2.9 ± 6.5*	0.000 *	*0.2 ± 5.6*	*0.843*
***∆BHb***	*0.2 ± 0.5*	0.002 **	*0.5 ± 1.1*	0.006 **	*0.6 ± 0.8*	0.000 ***	*0.2 ± 0.7*	0.001 *	*0.1 ± 0.7*	*0.239*
***∆BHp***	*0.1 ± 0.5*	*0.172*	*0.1 ± 0.7*	*0.620*	*0.2 ± 0.6*	0.037 *	*0.0 ± 0.6*	*0.677*	*0.4 ± 0.5*	0.000 ***
***∆aBTb***	*−0.5 ± 0.8*	0.000 ***	*−0.5 ± 0.9*	0.000 ***	*−0.8 ± 0.9*	0.000 *	*NA*	*NA*	*NA*	*NA*
***∆aBTp***	*−0.2 ± 0.9*	*0.183*	*−0.4 ± 0.9*	0.004 **	*−0.5 ± 0.9*	0.000 *	*NA*	*NA*	*NA*	*NA*
***∆mBTb***	*0.0 ± 0.3*	*0.552*	*−0.2 ± 0.3*	0.000 ***	*−0.4 ± 0.9*	0.011 *	*−0.3 ± 0.4*	0.000 *	*−0.1 ± 0.5*	*0.255*
***∆mBTp***	*−0.4 ± 0.5*	0.000 ***	*−0.3 ± 0.5*	0.000 ***	*−0.1 ± 0.6*	*0.225*	*−0.3 ± 0.5*	0.000 *	*0.0 ± 0.4*	*0.464*
***∆Root***	*−0.4 ± 0.6*	0.000 ***	*−0.4 ± 0.7*	0.000 ***	*0.0 ± 0.8*	*0.923*	*0.1 ± 0.9*	*0.144*	*−0.5 ± 0.9*	0.001 **

* *P* < 0.05, ** *P* < 0.01, *** *P* < 0.001, NA: not available.

**Table 3:** Mean values of the variable differences between pre- and post-treatment measurements for the mandibular arch.

*Teeth (mandible)*	*Central Incisors*		*Lateral Incisors*		*Canines*		*Premolars*		*First Molars*	
	*Mean ± SD*	*P*	*Mean ± SD*	*P*	*Mean ± SD*	*P*	*Mean ± SD*	*P*	*Mean ± SD*	*P*
***∆Tq***	*−0.3 ± 5.5*	*0.755*	*−2.1 ± 4.6*	0.004 *	*−4.1 ± 5.8*	0.000 ***	*−4.6 ± 4.5*	0.000 *	*−2.0 ± 7.1*	*0.070*
***∆BHb***	*0.5 ± 1.2*	0.005 **	*0.5 ± 1.1*	0.007 *	*0.6 ± 1.4*	0.007 **	*0.4 ± 1.1*	0.000 ***	*0.2 ± 0.6*	0.048 *
***∆BHp***	*0.3 ± 0.6*	0.002 **	*0.3 ± 0.7*	0.002 *	*0.2 ± 0.7*	*0.114*	*0.2 ± 0.6*	0.018 *	*0.2 ± 0.7*	*0.154*
***∆aBTb***	*−0.7 ± 1.2*	0.001 **	*−0.7 ± 1.0*	0.000 ***	*−1.0 ± 1.1*	0.000 ***	*NA*	*NA*	*NA*	*NA*
***∆aBTp***	*−0.5 ± 0.9*	0.000 ***	*−0.4 ± 1.1*	0.029 *	*−0.2 ± 0.9*	*0.269*	*NA*	*NA*	*NA*	*NA*
***∆mBTb***	*−0.3 ± 0.4*	0.000 ***	*−0.2 ± 0.4*	0.000 *	*−0.2 ± 0.5*	0.004 **	*−0.1 ± 0.4*	0.000 ***	*0.0 ± 0.4*	*0.609*
***∆mBTp***	*−0.3 ± 0.4*	0.000 ***	*−0.3 ± 0.6*	0.001 **	*−0.2 ± 0.6*	0.015 *	*−0.3 ± 0.7*	0.000 ***	*−0.1 ± 0.6*	*0.246*
***∆Root***	*−0.4 ± 0.7*	0.001 **	*−0.5 ± 0.8*	0.000 ***	*−0.1 ± 0.7*	*0.417*	*0.1 ± 1.1*	*0.586*	*−0.6 ± 0.5*	0.000 ***

* *P* < 0.05, ** *P* < 0.01, *** *P* < 0.001, NA: not available.

**Table 4:** Linear regression model for the torque variation of maxillary arch.

*Teeth (maxilla)*	*Central Incisors*		*Lateral Incisors*		*Canines*		*Premolars*		*First Molars*	
*Dependent Variables*	*R-square*	*P Value*	*R-square*	*P Value*	*R-square*	*P Value*	*R-square*	*P Value*	*R-square*	*P Value*
***∆aBTb***	*0.563*	0.000 ***	*0.249*	0.001 **	*0.520*	0.000 ***	*NA*	*NA*	*NA*	*NA*
***∆aBTp***	*0.622*	0.002 **	*0.256*	0.000 ***	*0.247*	0.001 **	*NA*	*NA*	*NA*	*NA*
***∆mBTb***	*0.061*	*0.720*	*0.111*	0.027 *	*0.094*	0.043 *	*0.026*	*0.131*	*0.072*	*0.082*
***∆mBTp***	*0.049*	*0.149*	*0.033*	*0.237*	*0.000*	*0.939*	*0.000*	*0.910*	*0.020*	*0.360*
***∆BHb***	*0.010*	*0.520*	*0.003*	*0.711*	*0.002*	*0.783*	*0.009*	*0.371*	*0.000*	*0.894*
***∆BHp***	*0.000*	*0.911*	*0.063*	*0.100*	*0.053*	*0.131*	*0.037*	*0.071*	*0.002*	*0.758*
***∆RootL***	*0.043*	*0.175*	*0.001*	*0.842*	*0.012*	*0.478*	*0.003*	*0.619*	*0.001*	*0.879*

* *P* < 0.05, ** *P* < 0.01, *** *P* < 0.001, NA: not available.

**Table 5:** Linear regression model for the torque variation of mandibular arch.

*Teeth (mandible)*	*Central Incisors*		*Lateral Incisors*		*Canines*		*Premolars*		*First Molars*	
*Dependent Variables*	*R-square*	*P Value*	*R-square*	*P Value*	*R-square*	*P Value*	*R-square*	*P Value*	*R-square*	*P Value*
***∆aBTb***	*0.733*	0.000 ***	*0.580*	0.004 **	*0.223*	0.001 **	*NA*	*NA*	*NA*	*NA*
***∆aBTp***	*0.613*	0.000 ***	*0.288*	0.000 ***	*0.062*	*0.104*	*NA*	*NA*	*NA*	*NA*
***∆mBTb***	*0.363*	0.000 ***	*0.269*	0.000 ***	*0.001*	*0.878*	*0.016*	*0.248*	*0.123*	0.02*
***∆mBTp***	*0.228*	0.001 **	*0.051*	*0.140*	*0.003*	*0.734*	*0.014*	*0.280*	*0.000*	*0.925*
***∆BHb***	*0.002*	*0.780*	*0.008*	*0.555*	*0.001*	*0.819*	*0.030*	*0.107*	*0.000*	*0.931*
***∆BHp***	*0.040*	*0.192*	*0.029*	*0.268*	*0.001*	*0.853*	*0.063*	0.019*	*0.076*	*0.070*
***∆RootL***	*0.098*	*0.039*	*0.001*	*0.818*	*0.008*	*0.575*	*0.000*	*0.904*	*0.035*	*0.223*

* *P* < 0.05, ** *P* < 0.01, *** *P* < 0.001, NA: not available.
